# The Potential of PET/MRI Imaging in Oncology: a Comment to a Summary Report of the First PET/MRI Workshop in Tuebingen in 2012

**DOI:** 10.1007/s11307-013-0642-y

**Published:** 2013-05-21

**Authors:** Johannes Czernin, Ken Herrmann

**Affiliations:** Ahmanson Translational Imaging Division, Department of Molecular and Medical Pharmacology, David Geffen School of Medicine at UCLA, 10833 Le Conte Avenue, Room 2-222 CHS, Los Angeles, CA 90095-1782 USA

A recent volume of Molecular Imaging and Biology includes a report of a workshop on preclinical and clinical positron emission tomography (PET)/magnetic resonance imaging (MRI). The workshop was prompted by the emergence of integrated clinical PET/MRI systems [1] and provided a forum for sharing initial experiences in the preclinical and clinical applications of this technology. The authors correctly point out that PET/MRI “must offer new areas of applications exploiting the strengths of each technology” and, as such, should not be in conflict with PET/computed tomography (CT).

The strength of PET imaging as the only mature clinical molecular imaging tool is evident. Its critically important role for initial and subsequent management decision in cancer patients is undisputed [2]. At the same time, MRI is emerging as a powerful tool to image biological processes including tumor perfusion, oxygenation, metabolism, and others (reviewed in [3, 4]). However, it is unknown whether the combination of PET and MRI in integrated PET/MRI systems improves patient management and outcome.

It is noteworthy that CT has remained the dominant force in cancer imaging despite significant expansion of MRI capabilities (with the notable exception of brain tumor imaging). The number of CT studies outnumbers that of MRI by factors of 4, 12, 5.5, 7.9, and 4 in breast, colorectal, lung cancer, non-Hodgkin lymphoma, and prostate cancer, respectively [5]. Simple explanations are that CT is well tested, safe, comparably inexpensive, easy to operate, and useful for managing cancer patients. In fact, almost every intervention contemplated in cancer patients (biopsy, surgery, radiation therapy) requires the use of CT imaging.

While it is premature to offer an appraisal of the role of clinical PET/MRI imaging, some theoretical advantages are evident. First, PET/MRI exposes patients to much less radiation than PET/CT. Second, MRI has been reported to detect and stage some cancers with a higher accuracy than CT. Finally, PET/MRI has the capability to obtain biological, functional, and anatomical data near simultaneously.

However, some of these proposed advantages warrant a critical examination. For instance, one well-publicized concern about CT is the associated radiation exposure of patients [6]. While this concern is appropriate when CT imaging is used inappropriately (for instance, for cancer screening), such risks are greatly diminished for cancer patients with a limited life expectancy [7]. Moreover, using different models, a more recent analysis suggested that “risks of medical imaging at effective doses below 50 mSv for single procedures or 100 mSv for multiple procedures over short time periods are too low to be detectable and may be nonexistent” and that “predictions of hypothetical cancer incidence and deaths in patient populations exposed to such low doses are highly speculative and should be discouraged” [8]. Nevertheless, reduced radiation exposure through the use of PET/MRI rather than PET/CT could be advantageous in “at risk” populations such as children and women of childbearing age.

Reports have suggested that MRI is more accurate than CT for several oncological indications including prostate cancer, pancreatic cancer, primary or metastatic liver cancer, bone metastasis, head and neck cancer, and others. However, the evidence in support of this notion is hard to find. For instance, in pancreatic cancer, CT and MRI are equally accurate, and the choice between modalities is frequently determined by the level of expertise and availability of the modality [9], and neither CT nor MRI detected vascular invasion reliably [10]. A meta-analyses for lymph node staging in head and neck cancer also failed to find improved accuracy of MRI over CT [11]. A meta-analysis in prostate cancer revealed low accuracies for both MRI and CT imaging [12]. However, using a lymph node-specific contrast agent improved the accuracy of MRI over CT [13]. In yet another meta-analysis, MRI and CT detected liver metastases with a comparable accuracy, and both tended to perform worse than 2-deoxy-2-[^18^F]fluoro-D-glucose (FDG) PET/CT [14]. The notable exception is the imaging of brain tumors for which MRI is clearly superior to CT. Thus, there is little evidence that MRI exceeds the accuracy of CT in any cancer other than brain tumors, and there is even less evidence that MRI is cost-effective in any setting including breast cancer [15] and pancreatic cancer [16].

The ability to simultaneously acquire molecular, functional, and anatomical information using PET/MRI has also been used as a strong argument for the use of PET/MRI (Bailey et al., workshop report). Conceptually, this ability is intriguing if one assumes that (a) biological processes change within minutes and (b) that therapeutic interventions elicit biological responses within the time frame of a combined PET/MRI study. For instance, tumor FDG uptake is relatively stable even if PET measurements are repeated within up to 1 week [17]. Moreover, MRI tumor perfusion measurements were reproducible within 20 % even when studies were performed 2–7 days apart [18].

The good reproducibility of PET and MRI measurements over several days suggests that simultaneous measurements may not be critically important for clinical application since the processes under study appear to be fairly stable.

There is, however, a clear advantage for PET/MRI to investigate, in research studies, molecular and functional processes simultaneously. From these research studies, insights might be gained that eventually could lead to important clinical applications.

As proposed by PERCIST [19], early partial metabolic tumor response is defined as a decrease in tumor FDG uptake by 30 %. Reductions in FDG uptake become more significant when time is allowed for additional cycles of chemotherapy. Since metabolic or functional tumor responses to treatment require at least several days to be measurable, it is quite unlikely that measurements of functional and molecular parameters are required to determine such changes simultaneously. The relative stability of metabolic and perfusion measurements from PET and MRI is not surprising. It is highly unlikely that gene expression, transcription, and translation, whether under baseline condition or in response to treatment, are executed within minutes or a few hours. Only such rapid changes in biological processes would require simultaneous measurements.

Many cancer patients undergo MRI as their primary imaging study in cancer. It clearly would be prudent to study these patients with PET/MRI and determine whether the integrated modality provides diagnostic, prognostic, or even predictive benefits over the individual imaging modalities.

MRI is a powerful imaging tool, and applications now include, among others, anatomical whole body surveys as well as measurements of metabolism, hemodynamics, vascularization, tumor viability apoptosis, and others [4]. However, only well-designed research studies will determine whether combining PET and MRI in a single imaging device provides incremental and translatable information that could not be obtained by stand-alone systems.
